# Downregulation of microRNA-145 may contribute to liver fibrosis in biliary atresia by targeting ADD3

**DOI:** 10.1371/journal.pone.0180896

**Published:** 2017-09-13

**Authors:** Yongqin Ye, Zhihan Li, Qi Feng, Zimin Chen, Zhouguang Wu, Jianyao Wang, Xiaoshuo Ye, Dahao Zhang, Lei Liu, Wei Gao, Lihui Zhang, Bin Wang

**Affiliations:** 1 Shantou University Medical College, Shantou, Guangdong, China; 2 Department of General Surgery, Shenzhen Children’s Hospital, Shenzhen, Guangdong, China; 3 Shenzhen Key Laboratory of Hepatobiliary Disease, Shenzhen Third People’s Hospital, Shenzhen, Guangdong, China; 4 Department of Organ Transplatation, Tianjin First Center Hospital, Tianjin, China; 5 Department of Traditional Chinese Medicine, Shenzhen Children’s Hospital, Shenzhen, Guangdong, China; Texas A&M University, UNITED STATES

## Abstract

**Background and objectives:**

Biliary atresia (BA) is a pediatric liver disease characterized by fibro-obliteration and obstruction of the extrahepatic biliary system, that invariably leads to cirrhosis and even death, if left untreated for extended time. However, its pathology and etiology still remained unknown. In this study, we tested the expression of adducin 3 (ADD3), the gene identified as a susceptibility gene in BA by GWAS, and uncovered its upstream regulatory microRNA in the pathogenesis of BA.

**Methods:**

In this study, 14 infants with BA and 14 infants with choledochal cyst (CC) were enrolled as experimental group and control group, respectively. ADD3 and microRNA-145 (miR-145) expression profiles in liver tissues of BA and CC were determined using qPCR. Luciferase reporter assay was performed to verify the direct interaction between miR-145-5p and ADD3 3’ Untranslated Regions (3’UTR). The Lentiviral vectors containing miR-145, miR-145-3p inhibitor, miR-145-5p inhibitor, empty vector were transfected into human hepatic stellate cell line (LX-2) to determine the functional effect of miR-145 on ADD3 expression at both mRNA and protein level.

**Results:**

MiR-145 was shown to be down-regulated in liver tissues of infants with BA compared to CC (*p* = 0.0267). ADD3, verified as a target of miR-145-5p, was shown to be overexpressed in infants with BA at the mRNA level (*p* = 0.0118). Transfection of lentiviruses containing miR-145 into LX-2 cells decreased the expression of ADD3 at both mRNA and protein level compared to negative control group, and suppressed the expression of p-Akt at protein level.

**Conclusions:**

Our study has shown that overexpressed ADD3 and downregulated miR-145 were detected in BA liver tissues. MiR-145-5p was confirmed to target ADD3 by luciferase reporter assay. The downregulation of miR-145 may contribute to liver fibrosis in BA by upregulating the expression of ADD3.

## Introduction

Biliary atresia (BA) is a devastating form of neonatal cholestasis characterized by rapidly progressive fibro-obliteration of the extrahepatic bile ducts within the first few weeks of life. It has a much higher incidence in Southeast Asia, like Chinese and Japanese compared to North America and Western Europe (1/9600 ~ 1/5300 vs. 1/16000) [1–6]. The golden treatment nowadays remains Kasai operation, i.e., hepatoportoenterostomy, however, its prognosis is poor and the disease would progress to end-stage cirrhosis in most children[7]. Its pathogenesis was not clear though fibrotic obstruction of extrahepatic bile ducts was more acceptable.

MicroRNAs are endogenous small (18–23 nucleotides in length), non-coding RNAs that function to downregulate target gene expression and stabilize specific mRNA translation through binding to the 3’-untranslated region (3’UTR) in target genes, participating in a variety cell growth processes[8, 9]. Therefore, abnormal miRNA expression may involve in the development of multiple diseases. Many studies have been conducted to investigate that miRNAs participate in liver fibrosis process through regulating profibrotic gene expression. Recently microRNA-145 (MiR-145) has been implicated in liver fibrosis by regulating matrix gene expression in smooth muscle cells, suppressing transforming growth factor-β (TGF-β) dependent extracellular matrix (ECM) accumulation and fibrosis[10].

Adducin 3, i.e. ADD3, a membrane skeletal protein that functions in assembling spectrin-actin network in red blood cells and regulating cell-cell adhesions in epithelial tissues, including liver and biliary tract[11, 12]. It is more highly expressed in fetal liver relative to adult liver[13]. Studies have shown that actin and myosin were highly-deposited surrounding biliary canaliculi in BA infants[14]. Garcia et al. first identified that ADD3 was a susceptibility gene of BA by genome-wide association study (GWAS) in Asians. Tsai et al. discovered the same phenomenon in Caucasian population by GWAS[15]. In our study, we found that ADD3 is differentially expressed in BA liver biopsy tissues compared to control groups through immunohistochemistry (IHC).

TargetScan was used to predict the possible target site for ADD3, and it showed that miR-145-5p, but not miR-145-3p, may bind to ADD3 through its 3’UTR. In order to identify whether miR-145-5p indeed has a direct interaction with ADD3 and regulates its expression and thus, contributes to liver fibrosis in biliary atresia, we synthesize 4 different lentiviruses, miR-145, miR-145-3p inhibitor, miR-145-5p inhibitor and empty vector without miR-145, respectively. We then performed luciferase reporter assay and lentiviral transfection, showing that the expression of ADD3 was regulated by miR-145-5p.

## Materials and methods

### Patients

Our study was approved by Ethics Committee on Human Research of the Faculty of the Shenzhen Children's Hospital and was conducted based on the principles expressed in the 1975 Declaration of Helsinki. From 2015 to 2016, 14 infants suffered from BA and 14 age-matched infants suffered from choledochal cyst (CC) with a normal liver function were enrolled in our study. All infants were diagnosed with laparoscopic bile duct exploration by the same surgical team at Shenzhen Children’s Hospital. Clinical information was retrospectively analyzed in Table 1. Liver biopsy tissues were obtained at the time of surgery with a written informed consent from all patients’ guardians.

**Table 1:** Clinical information in infants with BA and CC.

	BA	CC	*P*
Age	85.33±4.618	98.50±2.584	0.0801
Male	6	7	N/A
Female	8	7	N/A
ALP, IU/L	623.6.9±49.16	201.6±22.75	<0.0001
ALT, IU/L	165.1±19.53	28.13±3.388	0.0001
AST, IU/L	1242.8±26.95	21.00±2.315	<0.0001
TBIL, μmol/L	170.3±9.555	17.00±1.102	<0.0001
DBIL, μmol/L	90.36±4.575	2.325±0.3624	<0.0001
GGT, IU/L	665.6±39.84	24.25±3.821	<0.0001

**BA**, biliary atresia; **CC**, choledochal cyst; **ALP**, Alkaline phosphatase; **ALT**, alanine transaminase; **AST**, aspartate transaminase; **TBIL**, total bilirubin; **DBIL**, direct bilirubin; **GGT**, gamma-glutamyltransferase.

### RNA extraction and quantification

Total RNA was extracted from liver tissues of BA and CC obtained during the time of surgery by using MiniBEST Universal RNA extraction Kit (TaKaRa, Dalian, China). cDNA of ADD3 was synthesized from each sample using PrimeScript RT reagent Kit (TaKaRa, Dalian, China) while that of miR-145 was synthesized from each sample using MiR-X miRNA First-Strand Synthesis (Clontech, Japan). 2mg of cDNA was used for real-time PCR (qPCR) by using SYBR-Green Master Mix (TaKaRa) and Mir-X^™^ miRNA qRT-PCR SYBR^®^ Kit (Clontech) on an ABI 7500 thermocycler (Applied Biosystems) to quantify relative expression of ADD3 and miR-145, respectively. The qPCR reaction for ADD3 contained the following: 10 μl of SYBR^®^ Premix Ex Taq II (Tli RNaseH Plus), 0.8 μl of forward primer, 0.8 μl of reverse primer, 0.4μ of ROX Reference Dye II (50x), 2 μl of cDNA template, and 6 μl of H2O. The reaction program was set at 95°C for 30s, followed by 40 cycles at 95°C for 5s and 60°C for 34s, and the melt curve was included. All measurements were repeated in triplicate and the ratio for the mRNA of ADD3 was normalized to GADPH while miR-145 was normalized to U6. The primers of ADD3 and GADPH for qPCR were synthesized by Takara company (Dalian, China) with the sequences of the followings: ADD3: 5’-TAGAAAAGAGAAATAAGATTCGGGAACA-3’ (forward) and 5’- CAATTCCAGCAAGCAACTGAGA-3’ (reverse); GADPH: 5’-CGACAGTCAGCCGCATCTT-3’ (forward) and 5’-CCCCATGGTGTCTGAGCG-3’. The primers of U6 and miR-145, with assay ID numbers of 203907 and 204483, respectively, were synthesized by Exiqon Company (Denmark).

### Cell culture

The activation of hepatic stellate cells (HSC) were shown to be involved in the progression of hepatic fibrosis and LX-2 cells, immortalized human HSCs with a myofibroblast-like phenotype, were used in our study[16]. The HSC cell line was obtained from ATCC. All cells were cultured in Dulbecco’s modified Eagle's medium (DMEM, Corning, USA) supplemented with 10% fetal bovine serum Australia Source (FBS, Corning, USA) under 37°C containing 5% CO_2_ in a humidified atmosphere. It took about 2 days for the cells to grow to about 80% of the culture plate before the culture media were subcultured and replaced.

### Lentiviral vectors transfection

Lentiviral vectors containing miR-145, miR -145-3p inhibitor, miR -145-5p inhibitor, empty vectors without microRNAs, with a sequence of the following, GAAAGGACGAGGATCCCACCTTGTCCTCACGGTCCAGTTTTCCCAGGAATCCCTTAGATGCTAAGATGGGGATTCCTGGAAATACTGTTCTTGAGGTCATGGTTGAATTCTAGTTATTAA, GAAAGGACGAGGATCCACCGAGCGAGAGCGCTCGGTATAAGAACAGTATTTCCAGGAATCCATACGCTCGGTAGAGACCGAGCGTTTTTTGAATTCTAGTTATTAA, GAAAGGACGAGGATCCACCGAGCGAGAGCGCTCGGTATAAGGGATTCCTGGGAAAACTGGACATACGCTCGGTAGAGACCGAGCGTTTTTTGAATTCTAGTTATTAA, respectively, were all synthesized by Takara (Dalian, China). 24 hours before lentiviruses transfection, LX-2 cells were counted by Nexcelom Cellometer (USA), according to the manufacture’s instructions, and then were inoculated in a 6-well-plate at a density of about 5x10^5^ cells per well, followed by being incubated at 37°C in a humidified atmosphere of 5% CO_2_ with 2ml DMEM for 24 hours. LX-2 cells were transfected with 3x10^6^ ifu of above lentiviruses together with polybrene (10ug/ml, Santa Cruz Biotechnology) in five groups, miR-145, miR-145 plus miR-145-3p inhibitor, miR-145 plus miR-145-5p inhibitor, miR-145-3p inhibitor plus miR-145-5p inhibitor, empty vectors, to generate high expression level of miR-145-5p and miR-145-3p, only high level of miR-145-5p, only high level of miR-145-3p, low level of miR-145-5p and miR-145-3p, no miR-145 but empty vector, respectively. 72 hours after transfection, cells were lysed and RNA was extracted by using MiniBEST Universal RNA extraction Kit (TaKaRa, Dalian, China).

### Western blot analysis

Protein was extracted from previous LX-2 cells transfected with different lentiviruses, followed by immuoblotting with ADD3 specific antibody (Santa Cruz Biotechnology, CA) at a dilution of 1:500, and β-actin antibodies (Cell Signaling Technology, Shanghai), were used at a 1:10000 dilution.

### Luciferase reporter assay

3’UTR sequence of ADD3 was obtained from NCBI (NM_001121.3). The primers were synthesized by Takara (Dalian, China) with the following sequence: 5’-AACGAGCTCGCTAGCCTCGAGATAAAGTCTTTTTATAATTATTATTATAACAATGTGA-3’ (forward) and 5’-CTTGCATGCCTGCAGGTCGACTTTTTTGGCAAACAAAGTTACTTCA-3’ (reverse). By using Phanta^®^ Max Master Mix, qPCR was used to amplify 3’UTR of ADD3 in genome DNA, according to the manufacturer’s instructions. Agarose electrophoresis was done for the qPCR product and showed a clear single band, followed by gel extraction by using QIAquick^®^ Gel Extraction Kit (QIAGEN). By using ClonExpress^®^ II One Step Cloning Kit (Vazyme, China), the qPCR product was cloned into the pmirGLO Dual-Luciferase miRNA Target Expression Vector (Promega, USA) that was double digested by *Xho*I and *Sal*I (Thermo Scientific). The recombination products were then transformed in competent cells and plated in a plate well contained ampicillin, followed by being identified by agarose electrophoresis. The positive PCR bacteria colony were cultured in LB medium contained ampicillin at 37°C overnight. The plasmids were extracted by using Qiagen^®^ Plasmid Midi Kit (QIAGEN, Germany). These plasmids were transfected into 5 groups of LX-2 cells by using lipofectamine^®^ 2000 Reagent (Invitrogen). Lentiviral transfection was performed after the medium were changed 4~6 hours post-transfection. Cells were harvested for 72 hours after transfection. Luminescence was detected by using the Dual-Glo^®^ Luciferase Assay System (Promega, USA) according to the protocol. Data were obtained by normalization to the Renilla luminescence in order to control the differences in transfection efficiency of different groups. In order to roll out the possibilities that miR-145 could regulate the luciferase activities other than binding to ADD3 3’UTR, a plasmid containedmutations in thepredicted sites was constructed by using the StarMut Site-directed Mutagenesis Kit (GenStar), with a primer sequence: 5’-ctgaaagtttttcttttgtaaaacctctttcagggtcttcaagtgcacattgctacatcccccaatctgatctaccattg-3’ (forward) and 5’-caatggtagatcagattgggggatgtagcaatgtgcacttgaagaccctgaaagaggttttacaaaagaaaaactttcag-3’ (reverse) (S1 Table). Transformation, plate coating, and plasmid extraction were performed the same as previous procedures. The mutated plasmid was identified by dual-enzyme digestion and sequencing (S1 Fig).

### MiRNA target prediction

MiRanda and TargetScan (http://www.targetscan.org/vert_71/) were used to predict possible target genes of miRNA[17]. The binding free energy and miRNA-binding sites in target genes were then analyzed and calculated through the RNAhybrid Web site (http://bibiserv.techfak.uni-bielefeld.de/rnahybrid)[18].

### Immunohistochemistry

A total of 28 liver biopsy tissues, including 14 from biliary atresia and 14 from choledochal cysts, were obtained during surgery and were then fixed in formalin, embedded in paraffin, sectioned at 5um for per sample. All of the patients’ guardians provided written informed consent. This study protocol was approved by the Ethics Committee of Research of the Faculty of the Shenzhen Children’s Hospital. IHC for ADD3 was performed by using monoclonal anti-human ADD3 antibodies (sc-25733) purchased from Santa Cruz Biotechnology (Santa Cruz, CA). Cells stained with brown-stained cytoplasms were regarded as positive for ADD3 expression.

### Statistical analysis

All data were reported as mean±standard error of mean (SEM). Comparisons were made between two groups by Student’s *t*-test using Prism 6.0 (GraphPad Software Inc). *P*<0.05 was considered as statistically significant for all the statistical analyses.

## Results

### MiR-145 is predicted to target 3’UTR of ADD3

**Fig 1:**
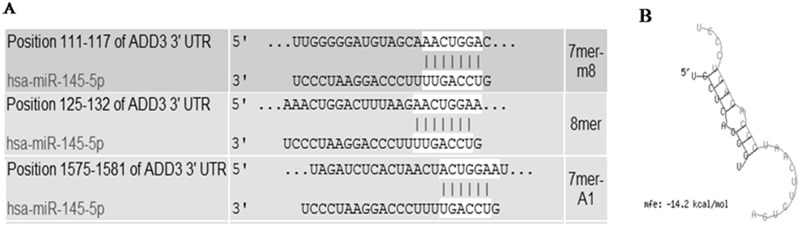
MiR-145 was predicted to target to ADD3 through 3’UTR interaction. (A) and (B) ADD3 was predicted to have a putative miR-145 binding site within its 3’UTR.

**Fig 2:**
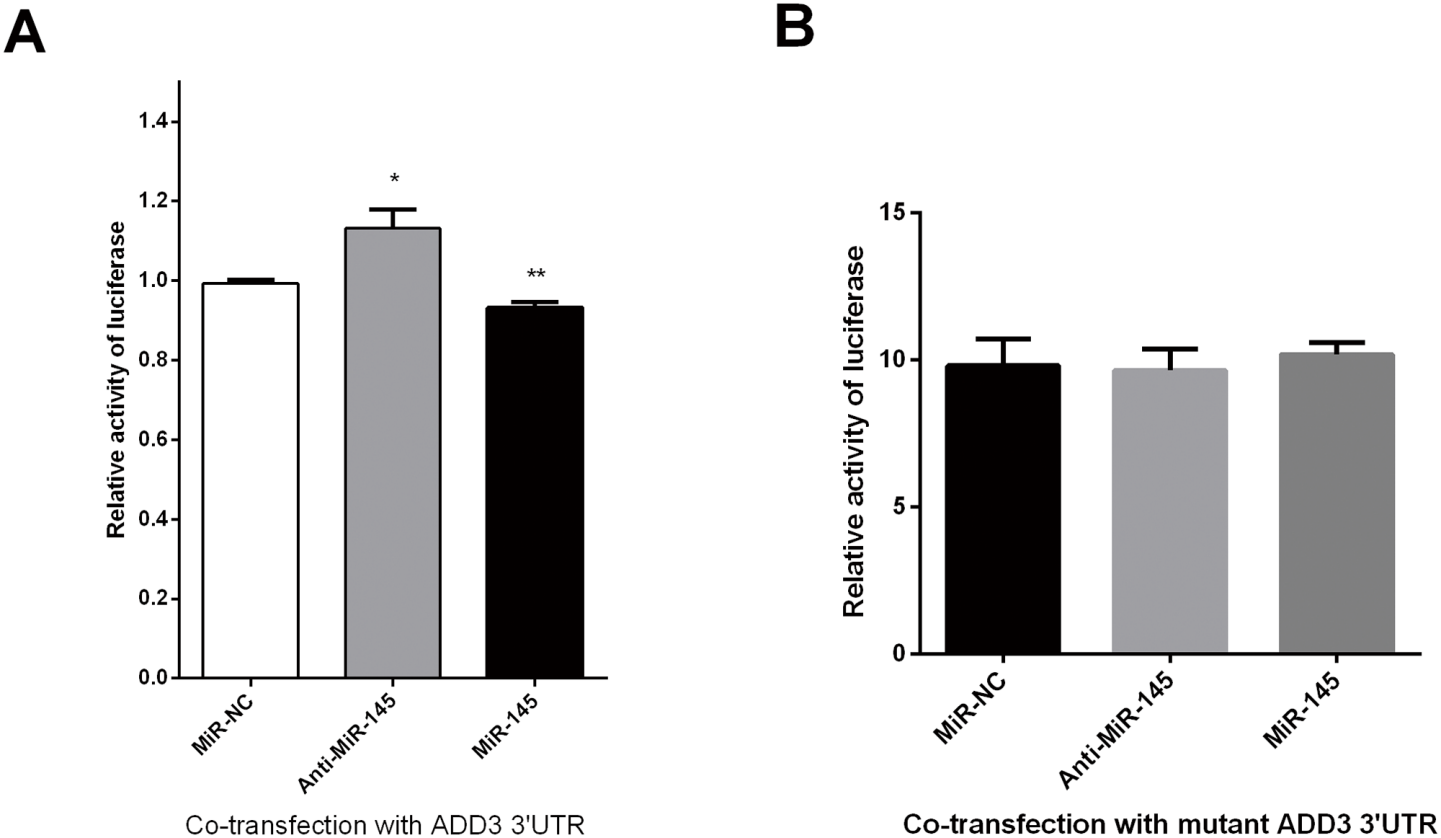
Luciferase reporter assays for miR-145 and ADD3 3’UTR co-transfection. (A) MiR-145 repressed the luciferase activity by binding directly to ADD3 3’UTR in LX-2 cells, indicating a direct interaction between miR-145 and ADD3 in LX-2 cells. (B) NO differential luciferase activities were seen among miR-145, anti-miR-145 and NC groups, indicating miR-145 exactly binds to ADD3. UTR = untranslated region.

To identify the potential targets of miR-145 in biliary atresia, MiRanda and TargetScan 7.1 were used. ADD3 was predicted to be a potential target of miR-145-5p in the base sequence of 111–117, 125–132 and 1575–1581, by binding to 3’UTR of ADD3 (Fig 1). It was as well confirmed by luciferase assays that miR-145 decrease the relative activity of luciferase by directly binding to 3’UTR of ADD3 in LX-2 cells (Fig 2A). A combined plasmid with mutations in the predicted binding site was generated and underwent co-transfection with different groups of miR-145 by luciferase assays, showing that there was NO significant difference among different groups for luciferase activities (Fig 2B).

### MiR-145 inhibits ADD3 expression in LX-2 cells

**Fig 3:**
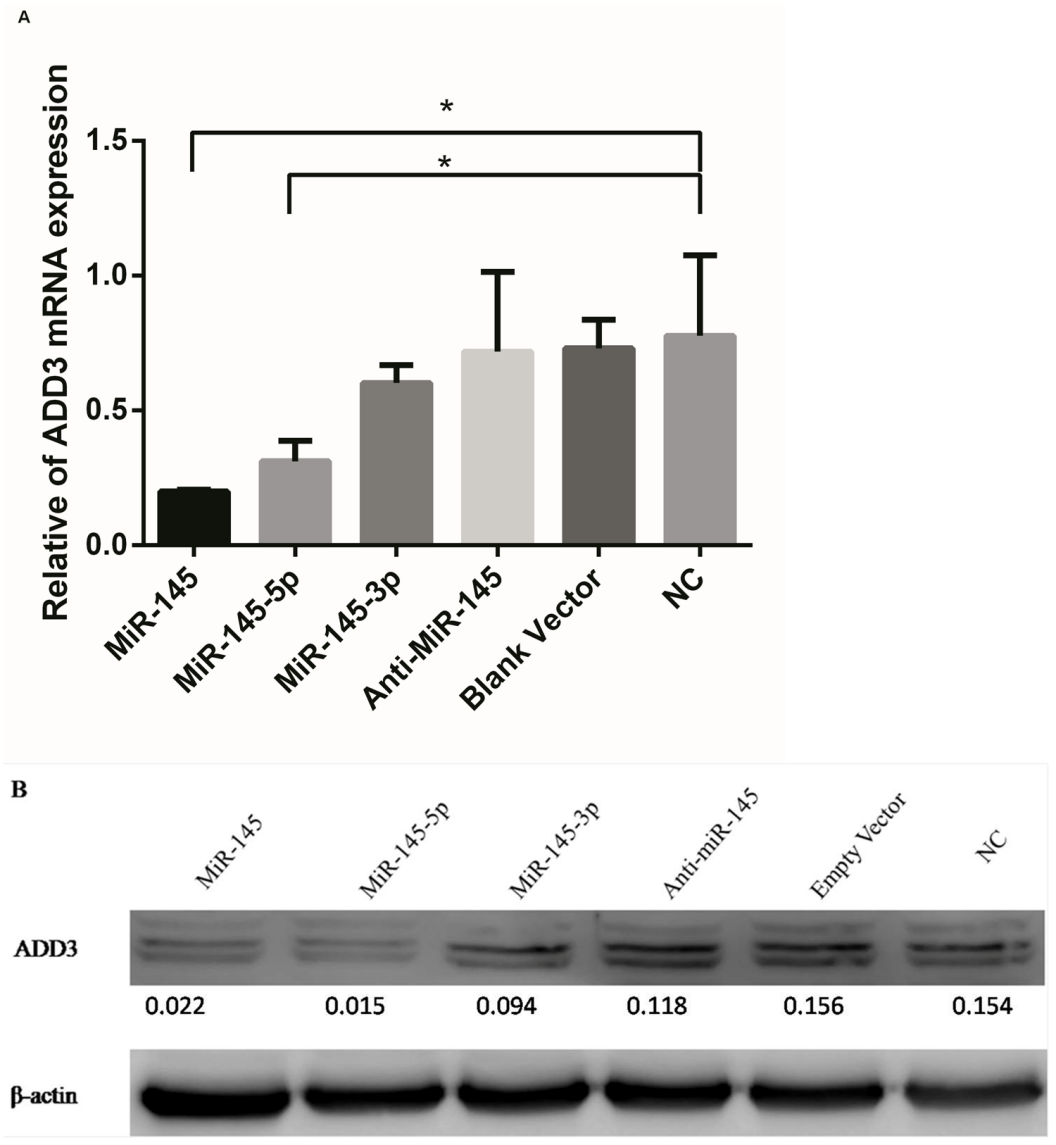
Expression of ADD3in LX-2 cells in the intervention of lentiviral transfection. (A) ADD3 mRNA was repressed byupregulated miR-145 transfection. (B) ADD3 protein expression was suppressed by miR-145.

**Fig 4:**
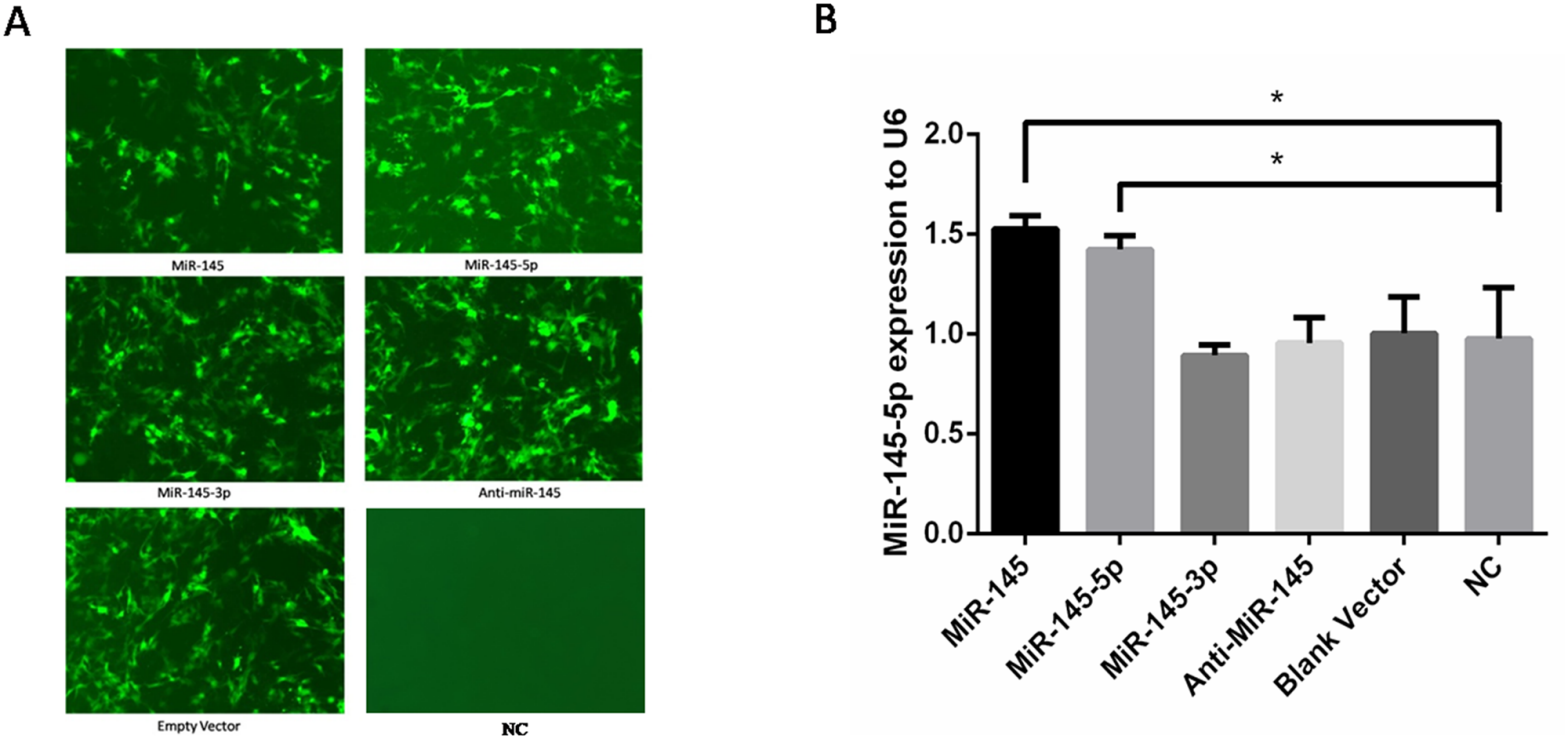
Evaluation of MiR-145 lentiviral transfection efficiency. (A) Green fluorescence was detected in each group of lentiviral transfection except NC group, indicating a successful transfection. (B) qPCR was performed to quantify the expression level of miR-145-5p in each group, showing an increased miR-145-5p in both group 1 and 2 while not significantly decreased miR-145-5p in anti-miR-145 groups, indicating a low basic level of miR-145-5p in LX-2 cells.

**Fig 5:**
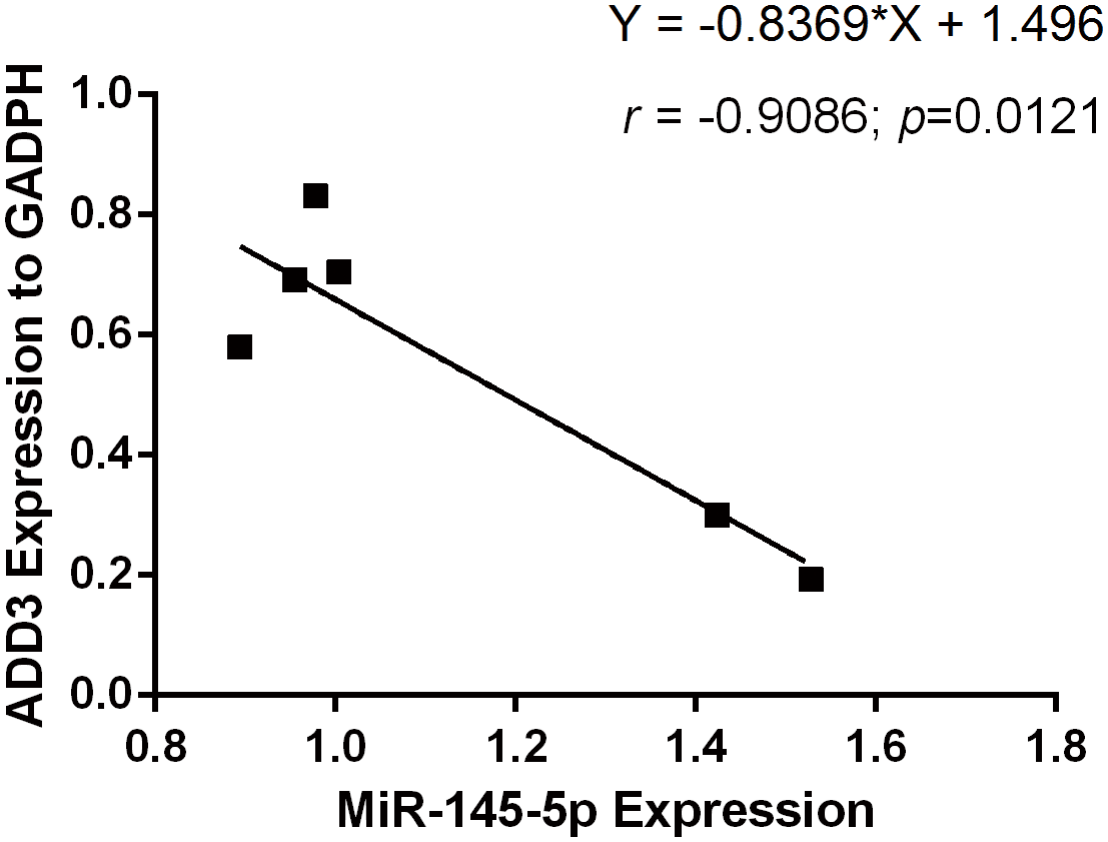
Pearson correlation analysis between miR-145-5p and ADD3. The expression level of ADD3 was shown to be negatively reated to miR-145-5p in LX-2 cell. Pearson r was -0.9086 and 95% confidence interval was between -0.9901 and -0.3693, with an equation of Y = -0.8369*X + 1.496. ρ = 0.0121.

MiR-145-5p was predicted to be broadly conserved while MiR-145-3p was very poorly conserved for binding to ADD3 3’UTR by TargetScan, taken together with the previous luciferase reporter assays, miR-145-5p was confirmed to target on ADD3 3’UTR. In order to further assess whether miR-145-5p but not miR-145-3p has a functional role in regulating endogenous ADD3 expression, 5 groups of lentiviral transfection intervention were built. Group 1: Increase both miR-145-5p and miR-145-3p by transfection with miR-145; Group 2: Only increase miR-145-5p by transfection with both miR-145 and miR-145-3p inhibitor; Group 3: Only increase miR-145-3p by transfection with both miR-145 and miR-145-5p inhibitor; Group 4: Decrease both miR-145-3p and miR-145-5p by transfection with both miR-145-3p inhibitor and miR-145-5p inhibitor; Group 5: Transfection with empty vector without miR-145 to control variables. Because miR-145-5p were both increased in LX-2 cells after transfection in group 1 and group 2, if it was miR-145-5p that had a more important role in regulating the expression of ADD3, then both groups would have a similar results. Similarly, if it was miR-145-3p that had an functional role in regulating ADD3 expression, ADD3 would not be repressed in group 2 but rather similar results would be shown in group 1 and group 3. qPCR was performed in 5 groups with lentiviral transfection plus 1 group without transfection to identify ADD3 mRNA expression level 72 hours after lentiviral transfection, which showed that miR-145-5p could suppress the expression of ADD3 mRNA as well as protein level. However, both group 3 and group 4, i.e. downregulated miR-145-5p didn’t significantly increase ADD3 expression at both mRNA and protein level compared to the NC group (Fig 3). In order to verify a successful lentiviral transfection, fluorescence was detected and qPCR for miR-145-5p was performed after transfection (Fig 4). Furthermore, correlation between miR-145-5p and ADD3 was analyzed by Pearson correlation analysis, showing that reduction of ADD3 expression was negatively related to the increase of miR-145-5p in vitro, with a 95% confidence interval of [-0.9901, -0.3693] and Pearson *r* of -0.9086 (Fig 5).

### MiR-145-5p inhibited Akt phosphorylation in LX-2 cells

**Fig 6:**
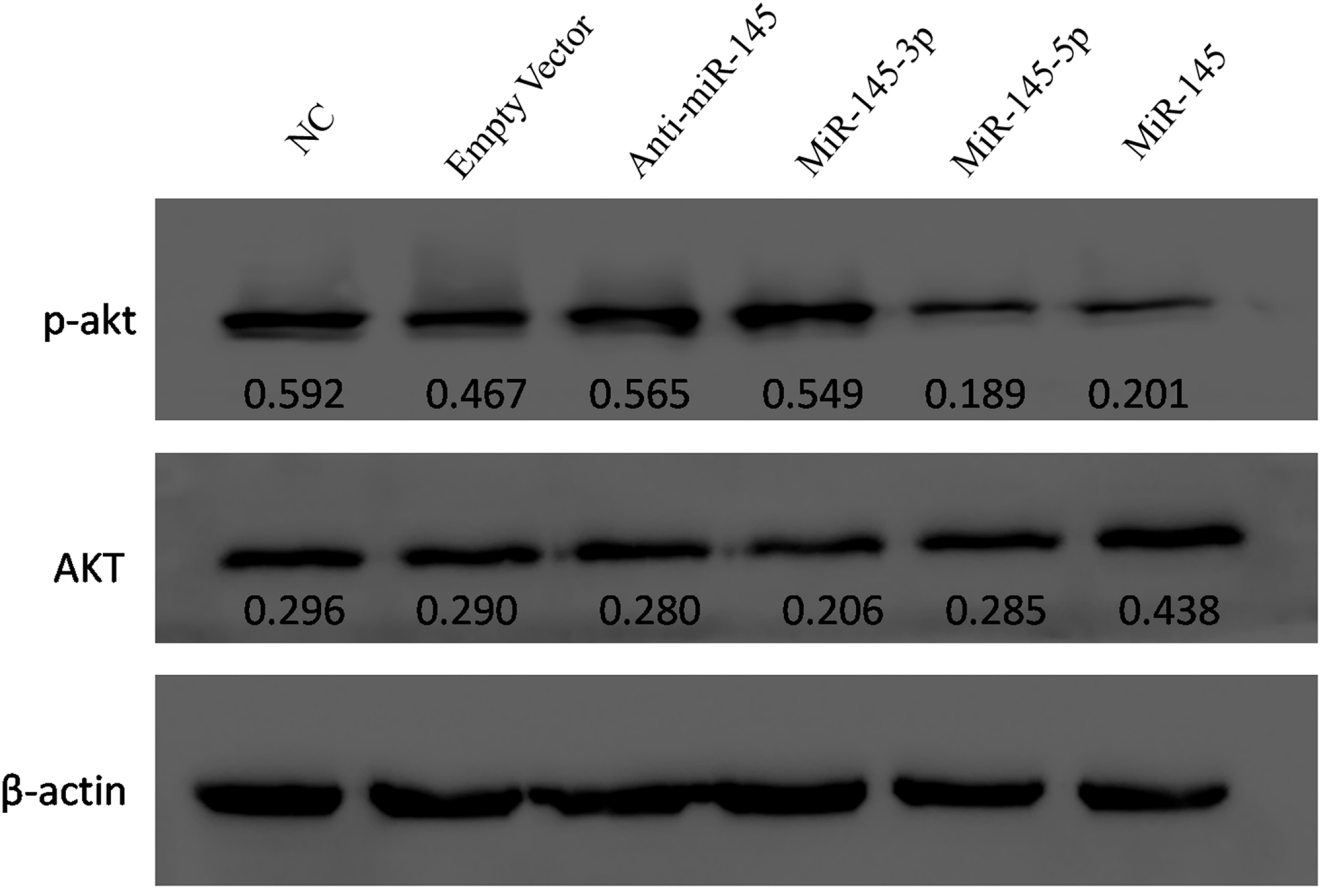
Evaluation of the effect of miR-145 on Akt phosphorylation by Western blot analysis. The protein levels of phosphorylated Akt (pAkt) was inhibited in both miR-145-5p and miR-145-transfected cells compared to NC groups.

Phosphorylated Akt is shown to be involved in pulmonary fibrosis and contribute to fibrogenesis, and many studies showed that increased Akt expression could cause advanced fibrosis[19, 20]. Therefore, we evaluated the effect of miR-145 on Akt phospohrylation. Akt phosphorylation was significantly inhibited in both miR-145 and miR-145-5p-transfected groups by Western blot analysis (Fig 6).

### ADD3 is overexpressed in BA liver tissues

**Fig 7:**
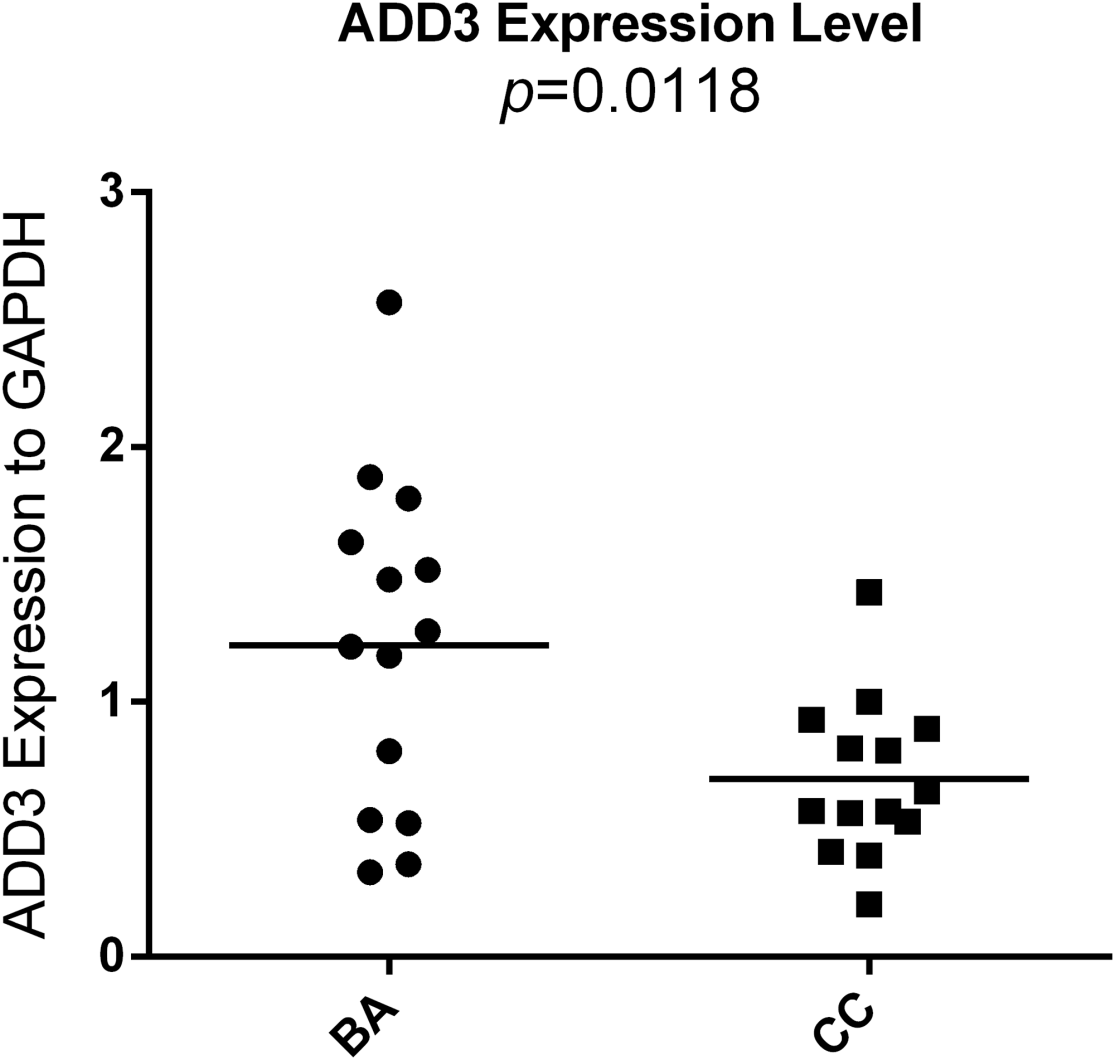
Expression of ADD3 at mRNA level in liver tissues. ADD3 mRNA was quantified in 14 infants suffered from BA and 14 infants suffered from CC by qPCR. Compared with CC controls, the expression of ADD3 was significantly higher in BA group (*p* = 0.0118).

**Fig 8:**
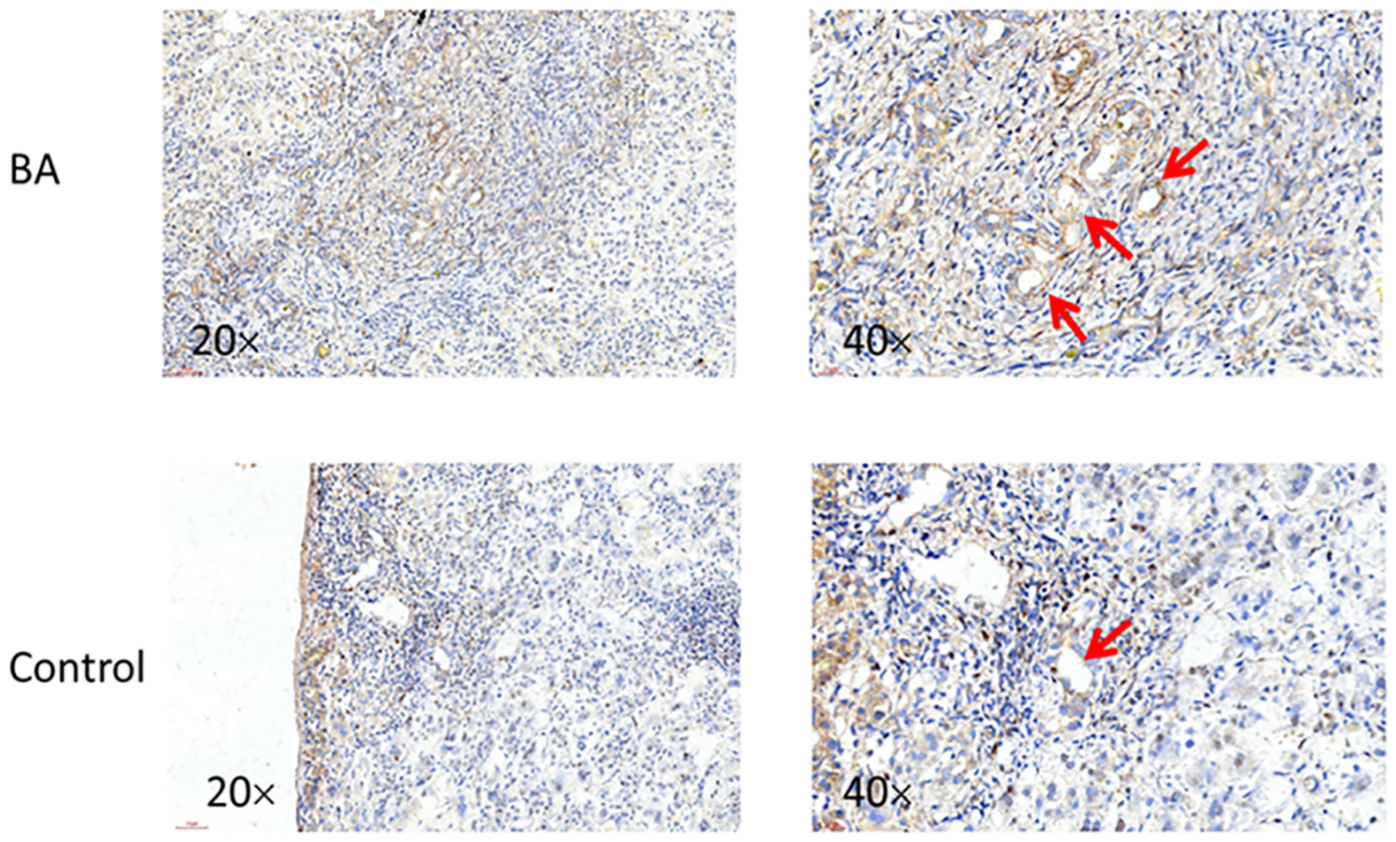
Immunohistochemistry analysis for ADD3 expression in BA and CC liver biopsy tissues. IHC for ADD3 was performed in BA and CC, showing a higher expression of ADD3 (red arrows) in BA group compared to CC group.

Total RNA of BA and CC were retracted from liver tissues during the surgery, followed by reverse transcription and qPCR. The mRNA expression of ADD3 were shown to be up-regulated in BA samples compared to that of CC (1.222 ± 0.1748 vs. 0.6987 ± 0.08251, *p*<0.05) (Fig 7). This result was confirmed by IHC for BA liver tissues (Fig 8).

### MiR-145 was downregulated in BA liver tissues

**Fig 9:**
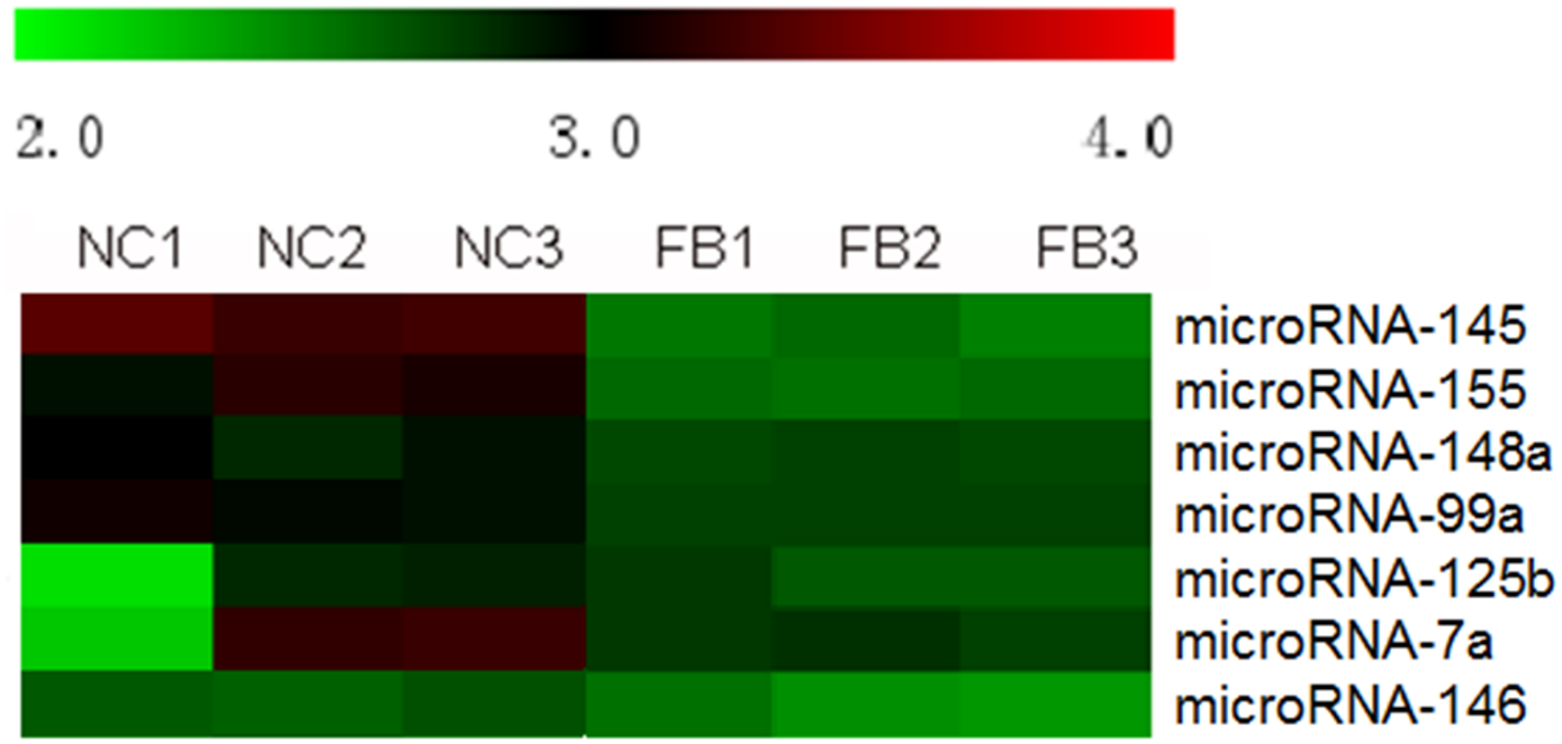
MicroRNA gene microarrays in liver tissues from both BA and NC. MiR-145 was shown to be differentially expressed in liver tissues of BA.

**Fig 10:**
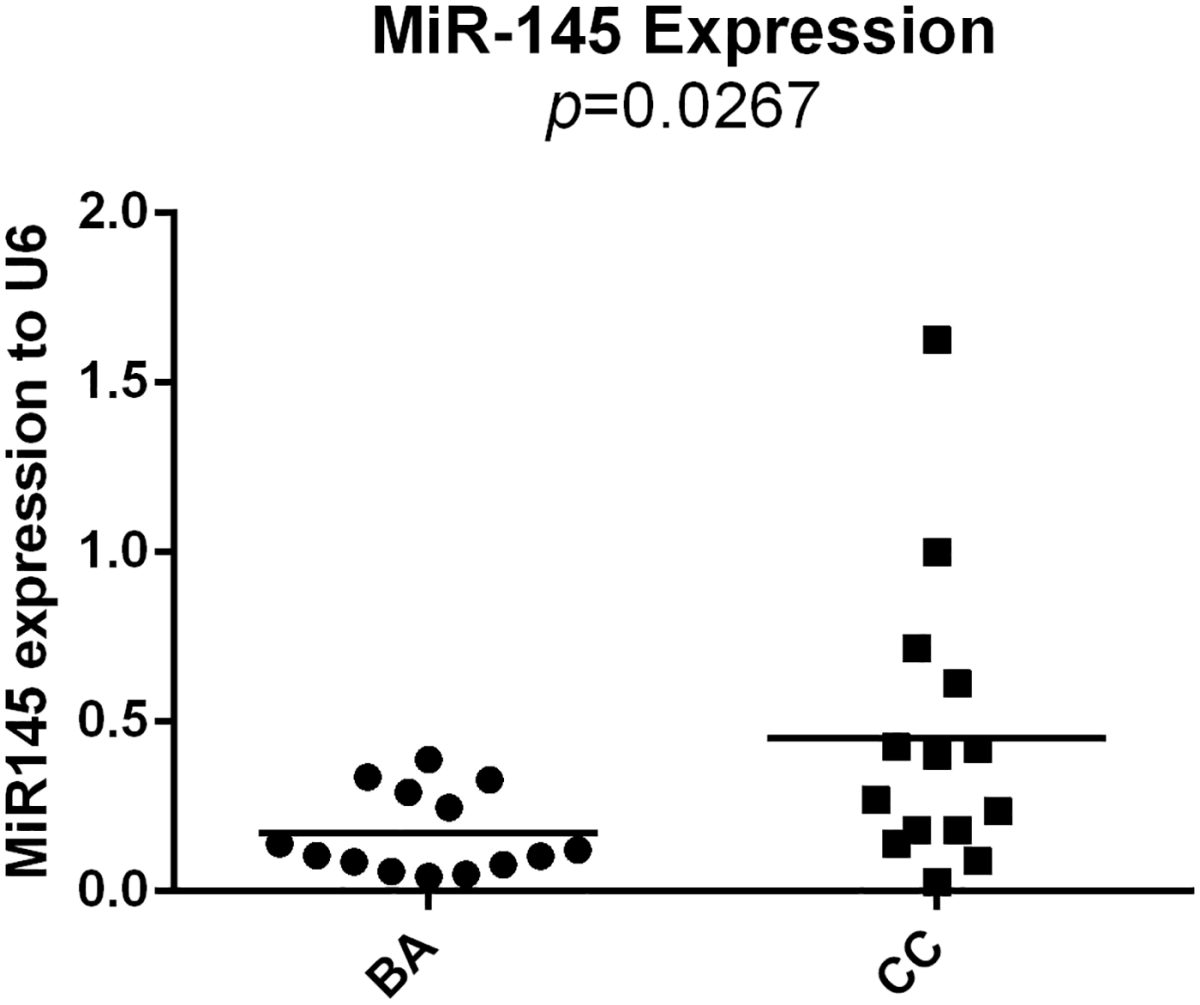
Expression level of miR-145-5p in BA liver tissues. qPCR was used to quantify the miR-145-5p expression in 14 infants with BA and 14 infants with CC. Compared with CC groups, the expression of miR-145 was significant in BA group (*p* = 0.0267)

In order to figure out the expression profile of microRNA in liver tissues suffering from BA, microRNA gene microarrays were once performed on 3 liver samples from BA and 3 liver samples from normal control. 31 differentially expressed of microRNAs were identified, including miR-145 (Fig 9). Furthermore, qPCR for miR-145 was performed. MiR-145-5p was used as a primer for qPCR reaction to verify the expression of MiR-145 in BA liver tissues. 14 infants with BA and 14 infants with CC were enrolled in the analysis, showing that expression of miR-145 was significant lower in BA group than that in CC group (0.1706 ± 0.03245 vs. 0.4518 ± 0.1152, *p*<0.05) (Fig 10).

## Discussion

Biliary atresia is a devastating neonatal cholangiopathy that destroys extrahepatic bile ducts and obstruct bile flow with an incidence rate of about 1/5300 in Taiwanese population[1]. It is one of the most common cause of end-stage liver disease and liver cirrhosis, and the main indication for liver transplantation in the United States[21, 22]. Early diagnosis of BA is essential for good outcome followed by surgical intervention of Kasai HPE surgery, because progressive liver fibrosis is commonly seen in BA and it is a key factor for prognosis. However, the specific etiology of fibrosis in BA is still in blur. Therefore, understanding of the pathogenesis and associated outcomes of BA is crucial for early diagnosis as well as treatment.

Garcia, et al. conducted a GWAS in Asian population and was the first to identify that ADD3 may be a susceptibility gene in BA, which was further confirmed by Tsai et al. through a replicated GWAS[15, 23]. Adducin γ, one of the expressed isoform from ADD3, is critical in spectrin-adducin-ankyrin complexes formation, which is the site that adducins regulate spectrin-actin cytoskeleton interactions[24]. Shtever et al. found a positive correlation between the intensity of alpha-smooth-muscle actin and the degree of liver fibrosis in BA infants[25].

Therefore, we collected liver tissues and analysed the expression of ADD3 in BA livers. Our analysis indicated a higher expression of ADD3 mRNA in BA liver tissues compared to the control groups at the mRNA level. Additionally, IHC revealed an increased ADD3 expression in BA liver biopsy samples compared to CC. As expected, these clinical data suggested that the dysregulated expression of ADD3 may contribute to BA. As overexpression of ADD3 could facilitate deposition of actin, adhesion and accumulation of epithelial cells, it may result in a poor contraction and poor bile flow, exacerbating cholestasis. This could cause severe accumulation of bile acids, aggravating liver fibrosis progress and promoting cholangiocyte proliferation, inducing obliteration of biliary tract[26].

MicroRNAs are endogenous small non-coding RNAs that function to downregulate the expression of target genes by binding to the specific sequences in their mRNAs[8, 27, 28]. To find out which miRNAs may target ADD3 and regulate its expression to induce liver fibrosis in BA, TargetScan was used in our study. The conservation analyses and sequence complementarity predicted by TargetScan showed that ADD3 3’UTR was broadly conserved sites for miR-145-5p. MiR-145 was found to destablize cell skeleton by binding to junctional adhesion molecule-A, which could guide inflammatory cells to disturbed flow in large arteries to develop artherosclerosis[29, 30]. Zhou et al. [10] found that miR-145 inhibited the activation and proliferation of hepatic stellate cells (HSCs) by binding to ZEB2, and thus suppress the progression of liver fibrosis. Therefore they suggest that miR-145 could be one of the treatments for liver fibrosis in BA infants. Moreover, in our microRNA gene microarrays, we figured out that miR-145 was differentially expressed in BA liver tissues. Therefore, we selected miR-145 as our study direction that miR-145-ADD3 may contribute to liver fibrosis in biliary atresia.

In order to verify this interaction, we performed luciferase reporter assay by cloning 3’UTR of human ADD3 into pmirGLO luciferase vector, followed by cotransfection with lentiviruses containing miR-145, anti-miR-145-5p and empty vector into LX-2 cells to identify their luciferase activities, respectively. It revealed that over-expressed miR-145 repressed the activity of luciferase with ADD3 3’UTR, which conformed to the predicted results of TargetScan. We as well generated a plasmid containing mutations in predicted miR-145-ADD3 sites. This mutant ADD3 3’UTR was co-transfected with different lentiviral groups as above, showing NO difference in luciferase activities among these groups. This further confirmed a direct interaction between miR-145-5p and ADD3.

To investigate whether miR-145 could regulate endogenous ADD3 expression *in vitro*, LX-2 cells, which belong to HSC cell line, were used in our study. The activation of HSCs plays an important role in the progression of liver fibrosis by promoting collagen and extracellular matrix synthesis and accumulation[31, 32]. Hepatic fibrosis is a key feature observed in infants with BA, though the specific mechanism still remains unknown.

Consistent with the results of luciferase reporter gene assay, both ADD3 mRNA and protein expression level were significantly suppressed by miR-145. Furthermore, Pearson correlation analysis was performed to evaluate the relationship between miR-145-5p and ADD3, which indicated that ADD3 was negatively related to miR-145-5p. However, when transfected with anti-miR-145, there was no significant increased expression of ADD3 neither at the mRNA nor protein level. We supposed that it was because LX-2 cells were related to fibrosis, and according to the findings from Zhou et al. [10]], miR-145 could suppress the proliferation of HSC and thus limit fibrosis progression. So the original level of miR-145 may be low in LX-2 cells, even though anti-miR-145 was transfected into LX-2 cells, there would be no further influence on ADD3. And this was confirmed in the qPCR for miR-145-5p after transfection.

In the intervention group 2 and group 3, i.e. overregulated miR-145-5p and overregulated miR-145-3p, we found that ADD3 mRNA were suppressed more in the former group although both were suppressed, suggesting that miR-145-5p may play an important role in regulating ADD3 mRNA expression. Similar results were seen in more obviously decreased ADD3 protein level in group 2 rather than group 3 by western blotting. Although downregulated miR-145-5p was supposed to increase ADD3 expression level, its original level of miR-145-5p in LX-2 cells may by low and thus unable to upregulate ADD3 expression. Taken together, we indicated that miR-145-5p, but not miR-145-3p, directly regulated the endogenous expression of ADD3 in LX-2 cells. The lower expression of miR-145-5p in BA liver tissues could facilitate the activation and proliferation of HSCs and the corresponding higher expression of ADD3 could facilitate the adhesion of epithelial cells, dysregulate spectrin-actin interaction and thus aggregate the normal bile flow causing cholestasis and accelerating the progression of liver fibrosis.

Phosphorylated Akt (p-Akt), also known as protein kinase B, is associated with multiple cellular processes by affecting cell proliferation and programmed cell death, etc. Research showed that an increased expression of p-Akt was involved in pulmonary fibrosis and could contribute to fibrogenesis, which was showed as the most significant factor associated with increased p-Akt expression by multivariate logistic repression analysis[19, 20]. In our study, the protein level of p-Akt was evaluated by Western blot, showing that p-Akt was repressed by miR-145. Therefore, fibrosis process may be inhibited via regulation of miR-145 on p-Akt. As both ADD3 and p-Akt were regulated by miR-145-5p in LX-2 cells, we suppose that ADD3 may play a role in Akt signaling pathway, and facilitate the activation and proliferation of LX-2 cells, and thus contributes to advanced liver fibrosis. However, it needs a further investigation.

## Conclusions

Our findings revealed that ADD3 was upregulated in liver tissues of BA while miR-145-5p was downregulated. Luciferase reporter gene assay showed that ADD3 is a direct target of miR-145-5p. The expression of ADD3 was affected by miR-145 or anti-miR-145 in LX-2 cells. Downregulated miR-145-5p may contribute to liver fibrosis in BA by up-regulating the endogenous expression of ADD3.

## Supporting information

S1 Table Primer sequences for qPCR and luciferase reporter assay. (DOC)

S1 Fig Sequencing for mutant ADD3 plasmid. (TIFF)

S1 Data Original data for qPCR, WB and luciferasse reporter assay. (ZIP)
