# Supplementary material for: Downregulation of microRNA-145 may contribute to liver fibrosis in biliary atresia by targeting ADD3
Source: PLoS One. 2017 Sep 13;12(9):e0180896. doi: 10.1371/journal.pone.0180896 (PMC5597134; doi:10.1371/journal.pone.0180896)
Supplement: S1 Table — (DOC) [file pone.0180896.s001.doc]

**Supplementary Table:S1**

Primer sequences:

| **Gene** | **5'–3' Primer Sequence** | **No. of bases** |
| --- | --- | --- |
| **qRT-PCR** |  |  |
| ADD3 Forward | TAGAAAAGAGAAATAAGATTCGGGAACA | 28 |
| ADD3 Reverse | CAATTCCAGCAAGCAACTGAGA | 22 |
|  |  |  |
| GAPDH Forward | CGACAGTCAGCCGCATCTT | 19 |
| GAPDH Reverse | CCCCATGGTGTCTGAGCG | 18 |
|  |  |  |
| U6 | #203907, Synthesized by Exiqon Company |  |
| miR-145 | #204483, Synthesized by Exiqon Company |  |
|  |  |  |
| **Luciferase Reporter (ADD3 3’UTR)** |  |  |
| Forward | AACGAGCTCGCTAGCCTCGAGATAAAGTCTTTTTATAATTATTATTATAACAATGTGA | 58 |
| Reverse | CTTGCATGCCTGCAGGTCGACTTTTTTGGCAAACAAAGTTACTTCA | 46 |
|  |  |  |
| Mutant ADD3 3’UTR |  |  |
| Forward | ctgaaagtttttcttttgtaaaacctctttcagggtcttcaagtgcacattgctacatcccccaatctgatctaccattg | 80 |
| Reverse | caatggtagatcagattgggggatgtagcaatgtgcacttgaagaccctgaaagaggttttacaaaagaaaaactttcag | 80 |
