# Supplementary figures and images for: Downregulation of microRNA-145 may contribute to liver fibrosis in biliary atresia by targeting ADD3
Source: PLoS One. 2017 Sep 13;12(9):e0180896. doi: 10.1371/journal.pone.0180896 (PMC5597134; doi:10.1371/journal.pone.0180896)

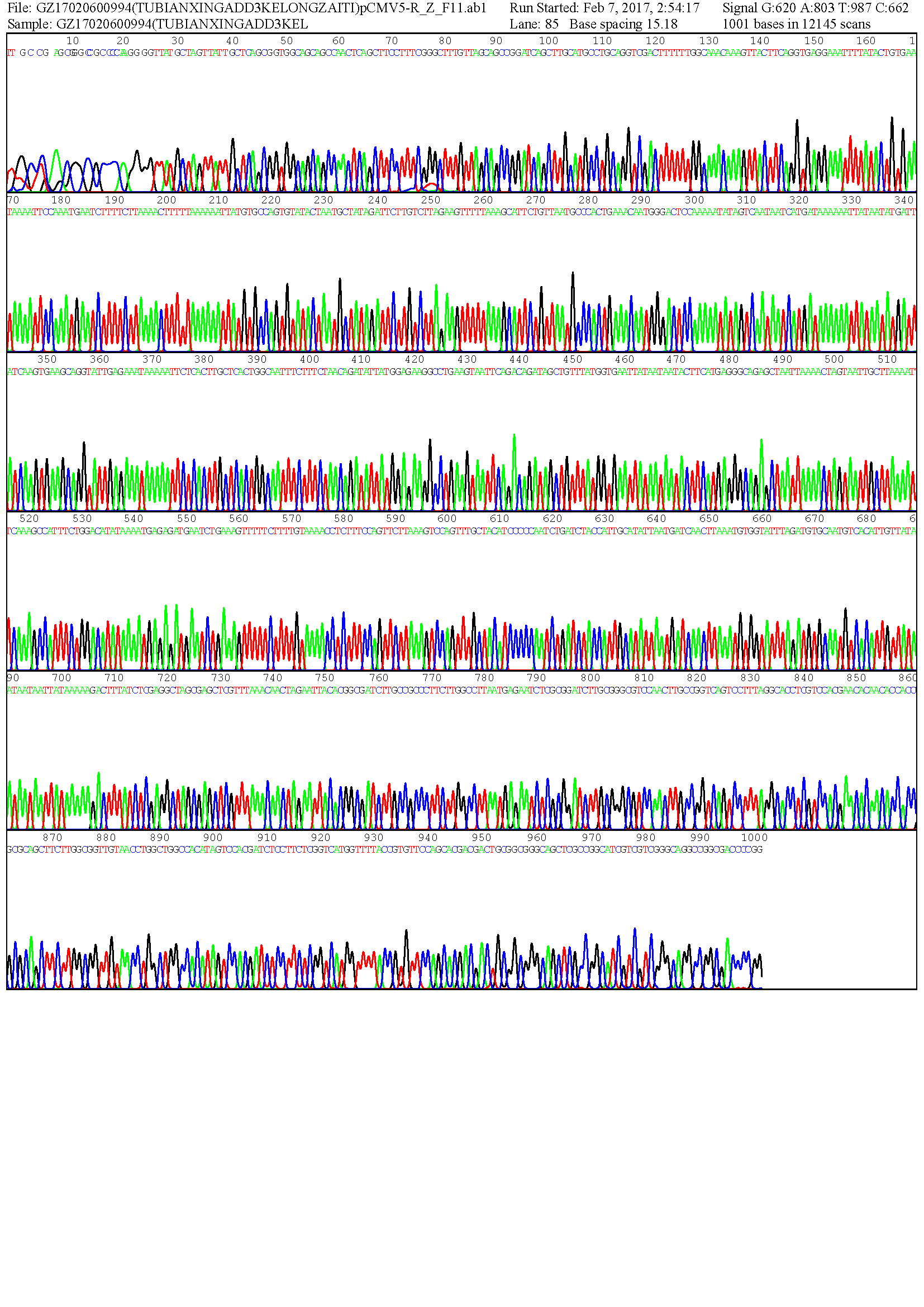

Supplement: S1 Fig — (TIFF) [file pone.0180896.s002.tiff]
